# Nuclear localization of Desmoplakin and its involvement in telomere maintenance

**DOI:** 10.7150/ijbs.34450

**Published:** 2019-08-24

**Authors:** Peipei Li, Yuan Meng, Yuan Wang, Jingjing Li, Manting Lam, Li Wang, Li-jun Di

**Affiliations:** 1 Cancer Center, Faculty of Health Sciences, University of Macau, Macau, SAR of China.; 2 Metabolomics Core, Faculty of Health Sciences, University of Macau, Macau, SAR of China.

**Keywords:** CRISPR BioID telomere DSP

## Abstract

The interaction between genomic DNA and protein fundamentally determines the activity and the function of DNA elements. Capturing the protein complex and identifying the proteins associated with a specific DNA locus is difficult. Herein, we employed CRISPR, the well-known gene-targeting tool in combination with the proximity-dependent labeling tool BioID to capture a specific genome locus associated proteins and to uncover the novel functions of these proteins. By applying this research tool on telomeres, we identified DSP, out of many others, as a convincing telomere binding protein validated by both biochemical and cell-biological approaches. We also provide evidence to demonstrate that the C-terminal domain of DSP is required for its binding to telomere after translocating to the nucleus mediated by NLS sequence of DSP. In addition, we found that the telomere binding of DSP is telomere length dependent as hTERT inhibition or knockdown caused a decrease of telomere length and diminished DSP binding to the telomere. Knockdown of TRF2 also negatively influenced DSP binding to the telomere. Functionally, loss of DSP resulted in the shortened telomere DNA and induced the DNA damage response and cell apoptosis. In conclusion, our studies identified DSP as a novel potential telomere binding protein and highlighted its role in protecting against telomere DNA damage and resultant cell apoptosis.

## Introduction

DNA-protein interaction is the fundamental biological activity in the nucleus. Understanding the underlying details of the DNA-protein interactions is very important to interpret the genetic information coded in the DNA sequence 1, 2. Previous studies have investigated the nuclear protein activities mainly through two biochemical approaches. The first one was trying to resolve the composition of the protein complexes that are functionally independent units. Many of these complexes are required for critical nuclear activities such as DNA replication, gene transcription etc.^3^, ^4^. The second approach was to determine the genome-wide binding profile of each individual protein such as the transcriptional factors through ChIP (chromatin immunoprecipitation) followed by deep sequencing 5. So far, hundreds of proteins have been examined using this approach under different physiological conditions in both tissues and cells, which greatly contributed to our understanding of the interaction between the proteins and chromatin, and particularly the interaction relevant gene transcriptional regulation 6. However, an emerging issue that dozens, if not hundreds, of proteins dynamically bind to the same DNA region comes together with a challenging question which is how to preserve the real-time binding activities of these proteins at a specific DNA locus and uncover the identities of these proteins simultaneously.

DNA-protein interaction is driven by the pioneer factors possessing DNA binding domains. Through protein-protein interaction (PPI), other proteins are recruited to the same DNA sequence by directly or indirectly interacting with the pioneer factors. The techniques to investigate the PPIs are quite diversified 7-13. To discover the stable PPIs, a common procedure is to pull down the target protein through immunoprecipitation and the co-precipitated proteins could be recognized by the non-targeted mass spectrometry 10, 14, 15. *In vivo*, the yeast or mammalian two/three hybridization system has been widely applied to identify novel PPIs 8, 16. For the transient PPIs, many developed approaches such as FRET, BRET and BiFC were applied to confirm the PPIs 12, 17-21. To capture the novel PPI *in vivo*, the proximity-dependent labeling (PDL) technologies were also developed that can be applied to analyze both stable and transient PPIs 22. The representative PDL approach is known as BioID 23. Theoretically, the bait protein is fused to an enzyme BirA* which has the capability to produce a reactive molecule 5'-AMP-Biotin at its reaction center domain with the presence of Biotin. Since the diffusion distance of 5'-AMP-Biotin is about 10-20 nm and the lysine residues of any protein within this distance have a chance to be biotinylated, the bait protein, together with its interacting proteins are the nearest target proteins of this biotinylation reaction 22, 24, 25. Therefore, the proteins that interact with the bait protein can be recognized through non-targeted mass spectrometry once the biotinylated proteins are captured by the streptavidin beads.

CRISPR/CAS9 is a genome-targeting tool and have been widely applied in genomic DNA editing 26. The essential components of CRISPR/CAS9 include the DNA endonuclease CAS9 that cleaves the target sequence and a small guide RNA (sgRNA) which recruits CAS9 to the target genome DNA. dCAS9 was developed by inactivating the endonuclease activity of CAS9. Fusion of dCAS9 with other proteins enables the recruitment of these proteins to the specific DNA sequence guided by sgRNA. For example, the epigenetic modification enzyme can be fused with dCAS9. With the presence of sgRNA targeting a specific genomic region, the fused enzyme can be recruited to this region to epigenetically modify the target DNA sequence 27, 28. Similarly, by fusing dCAS9 to fluorescent proteins, CRISPR/dCAS9 has been developed as a useful imaging tool 29. For instance, both the telomeres and centromeres could be imaged using dual color CRISPR/dCAS9 29. A recent attempt was to fuse dCAS9 with BirA* to create a novel technology CASID, which was applied to analyze binding proteins of highly repeated genomic regions 30.

A telomere is the chromatin region locating at the end of each chromosome and plays a crucial role in maintaining the structural and functional integrity of chromosomes. Human telomere DNA is a long double-strand, repetitive TTAGGG sequence followed by a few hundreds of single strand 3'-overhangs 31. In proliferating cells such as the stem cells, the maintenance of telomere DNA length requires the activity of hTERT (human Telomerase Reverse Transcriptase). In more than 85% of human cancers, reactivation of hTERT associates with the unusual extension of the telomere DNA in cancer cells 32. In most of the cells, the maintenance of telomere DNA length relies on the shelterin complex composed of TRF1, TRF2, POT1, TIN2, RAP1, and TPP1 etc. The shelterin complex is essential in preventing telomere DNA from nucleases approaching 31.In addition to the shelterin complex, some other proteins such as CST complex, TZAP and NuRD complexes were recently reported to be telomere-associated proteins 33-35. More large-scale studies were also recently published to investigate the telomere binding proteins 36-38.

Desmoplakin (DSP) is a component of desmosomes, the cell-cell adhesion structure on the cell membrane. Through its N-terminal domain and C-terminal domain respectively, DSP interacts with both the cadherin/plakoglobin/plakophilin complex and the intermediate filament (IF) cytoskeleton. Thus, DSP has mainly known as a cell membrane-associated protein. Loss of DSP results in the damage of the mechanical integrity of desmosomes and leads to diseases such as cardiovascular dysfunctions. However, DSP was also found to be a nuclear protein in our study when we performed CASID by fusing dCAS9 to BirA* and targeting the fused protein to the telomere. Moreover, DSP seems to bind to telomere through its C-terminal domain and to be indispensable in maintaining the structural and functional integrity of telomere.

## Experimental Procedures

### Constructs and antibodies

BirA*-dCas9-EGFP construct was generated by sub-cloning BirA*-HA from pcDNA3.1 MCS-BirA (R118G)-HA (Addgene #36047) into pSLQ1658-dCas9-EGFP (Addgene #51023), BglII and NCOI were selected restriction enzyme digestion sites. SgRNA-Telo construct was created by subcloning telomere repeat sequence into gRNA_Cloning Vector (Addgene #41824) with Gibson Assembly assay. To construct the DSP^∆NLS^, N-DSP, and C-DSP expression vectors, gene fragments were generated from 1136-Desmoplakin-GFP (Addgene #32227) by PCR, then the PCR fragments were cloned into pLJM1-EGFP. All the antibodies used in this research were as follow: anti-GFP (MBL, M048-3), anti-TRF1 (Santa cruz, sc-56807), anti-POT1 (Santa cruz, sc-81711), anti-TRF2 (Novus Biologicals, NB110-57130), anti-γH2AX(EMD Millipore, 05-636), anti-DSP (Abcam, 118804), anti-Phospho-Chk1 (Ser345) (Cell Signaling technology, #2341). Telo-sgRNA sequence: AGGGTTAGGGTTAGGGTTA.

### Cell culture and transfection

Human embryonic kidney cell line HEK293, human bone osteosarcoma epithelial cell line U2OS, human breast cancer cell line MDA-MB-231 and MCF-7 cells were cultured in Dulbecco's modified Eagle medium (DMEM, Gibco) with 2mM glutamine (Gibco) in 10% fetal bovine serum FBS (Gibco). All cells were maintained at 37℃ and 5% CO_2_ in a humidified incubator. For HEK293 cells, plasmid transfections were carried out using PEI, for other cell lines, plasmids were transfected with Lipofectamine 3000 (Thermo Fisher Scientific) according to the manufacturer's instructions.

### ShRNA and lentivirus infection

TRF1, TRF2, and DSP shRNA constructs were generated using pLKO.1TRC Cloning Vector (Addgene#10878), To produce shRNA lentivirus, HEK293FT cells were transfected with psPAX2, pMD2.G, and lentiviral plasmid pLKO.1-TRF1 shRNA(TRCN0000040161, TRCN0000130146), TRF2 shRNA (TRCN0000004811, TRCN0000155836), DSP shRNA (TRCN0000116409, TRCN0000116407), hTERT shRNA (TRCN0000219794, TRCN0000219795) or pLKO.1-scramble using PEI. Culture medium was collected 24 h and 72 h post-transfection and passed through 0.45μmfilters. The aliquot lentiviruses were stored at -80 °C. HEK293, MDA-MB-231andU2OS cells were infected by shRNA lentiviruses with polybrene. After 72 h, puromycin was added into the medium. For the infected cells, pools of puromycin-resistant cells were used. QPCR or Western blot was performed to screen for gene expression.

### Detection of biotinylated proteins and BioID pull down

Cells were seeded into a 6-well plate for growing until the cells were ready for transfection by the following vectors: empty vector, dCas9-EGFP-BirA* vector. 50μM biotin was added into the culture medium 3h post-transfection. 24 h later, the 6-well plate cultured cells were harvested for immunoblot assay. The cells were lysed by 100 μL cell lysis buffer (50mM Tris (pH 7.4), 150mM NaCl, 1%TritonX-100, 1% sodium deoxycholate, 0.1% SDS) on ice for 30 min with the presence of protease inhibitors, and further sonicated (40% output, 30s on, 30s off, 10 cycle) and centrifuged at 15,000g for 10 min at 4℃. These samples were analyzed by SDS-PAGE electrophoresis and protein was detected by streptavidin-HRP (Sigma).

### LC-MS

The protein-bound beads after pull-down were washed twice by 50 mM ammonium bicarbonate solution. 200 ul of 50 mM ammonium bicarbonate was used to suspend the beads. 7. 4 uL of 0.5 M Tris (2-carboxyethyl) phosphine (final concentration 10 mM) was added to the suspension and mixed for 30 minutes at 40°C. Then 8.8 uL of 0.5 M iodoacetamide (final concentration 20 mM) was applied to isolated proteins at room temperature in dark for 30 min. MS-grade trypsin (1:20 ratio, about 1 ug) was applied to digest the proteins for 16-18h at 37°C. Next, digested peptides were separated from magnetic beads by centrifugation, and adjusted to contain 0.1% TFA. The peptide solutions were cleaned up using C18 ziptip according to the manufacturer instruction. 3.5 μL of 30% ACN/0.1% TFA was applied to elute the proteins from ziptip by pipetting up and down for 20 times. 18 μL of 0.1% formic acid was added into each sample by pipetting up and down, then transferred to an HPLC vial. Finally, 15 μL of sample was carried out with LC-MS analysis (LC, UltimateTM3000 RSLCnano system (thermos Fisher Scientific); MS, Q-exactive TM quadrupole orbitrap mass spectrometer) and protein ID search using shotgun approach. The mass spectrometric data analysis was conducted by using the Peaks Studio 8.5 build 20171002, the sequence search was analyzed by Swiss-Prot database 2016_02 2016-02-17. Max missed tryptic cleavages was two, and one non-specific cleavage was allowed. Fragment mass tolerance for precursor ions and MS/MS fragments were 10 ppm and 0.05 Da, respectively. FDR of Peptide-Spectrum Matches, Peptide Sequences and Protein is no more than 1.0%.

### Immunofluorescence staining

Cells were seeded into an 8-well chamber for cell culture, and plasmids were transfected into the cells using PEI or Lipofectamine 3000. After 48-72 h post-transfection, the cells were fixed for 10 min in 4% paraformaldehyde at room temperature and were permeated with 0.2% TritonX-100 for 10 min. Then the cells were blocked with 1% BSA for 0.5 h, followed by primary antibodies incubation for 1 hour at room temperature. Then the chamber was washed in PBST three times and incubated with secondary antibodies for 30min. The secondary antibodies include Alexa 488,Alexa 594and streptavidin-Alexa Fluor (Life Technologies). Cells were counterstained with DAPI, and the chamber was mounted by anti-fade mounting medium (Beyotime). Finally, the fluorescence was observed by microscope.

### PNA FISH and Immunofluorescence with PNA FISH

For PNA FISH, cells were cultured in a 8-well chamber, the chamber was fixed in methanol:acetic acid (3:1) for 10 min at room temperature and washed in PBS for three time. Next the chamber was immersed in 4% paraformaldehyde for 10 min at room temperature. After fixation, the chamber was dehydrated using a series of cold ethanol washes (70%, 80%and 100%) and air dried for 10 min. The 500 nM of labeled PNA probe (TelC-Cy3 probe, Panagene) in 70% formamide, 1% (w/v) blocking reagent and 10 mM Tris, pH 7.2 was added into chamber and DNA was denatured at 80℃ for 3 min. Then the chamber was incubated in the dark at 37℃ for 1h. After hybridization, the chamber was washed with 70% formamide, 10 mM Tris pH 7.2 for 10 min and with TNT (0.05 M Tris/0.15 M NaCl/ 0.05% Tween-20, pH 7.5) for 10 min. Cells were counterstained with DAPI, and chamber was mounted by antifade mounting medium (Beyotime).

For IF-PNA FISH, after the incubation of primary antibody and secondary antibody, the chamber was dehydrated using a series of cold ethanol washes (70%, 80%and 100%) and air-dried for 10 min. Followed with the FISH steps and image analysis.

### Telomere-ChIP and Dot blot

The telomere-ChIP and Dot blot are according to Yang et al. 39 and the details of the procedure are provided in supplementary data.

### Telomere length measurement assay by Southern blot

Telomere length was measured by Telo TAGGG Telomere Length Assay Kit (Roche, 12209136001), the procedure was described in the manual. Briefly, genomic DNA was digested by HinfI and RsaI, the products were performed gel electrophoresis and membrane transfer. The membrane was hybridized with Dig-labeled telomere (TTAGGG)_3_ and detected by chemiluminescence system.

### Telomere length measurement by QPCR

Telomere length measurement by qPCR was performed as described previously^40^.

### Statistics

All the experiments were conducted at least three times and results within each experiment are described using mean ± S.E. Statistically significant (*p < 0.05, **p < 0.01, ***p < 0.001) were determined by using the two-sample Student's *t*-test. For western blotting and dot blotting, only the representative images were selected.

## Results

### CASID targeting telomere

**Figure 1:**
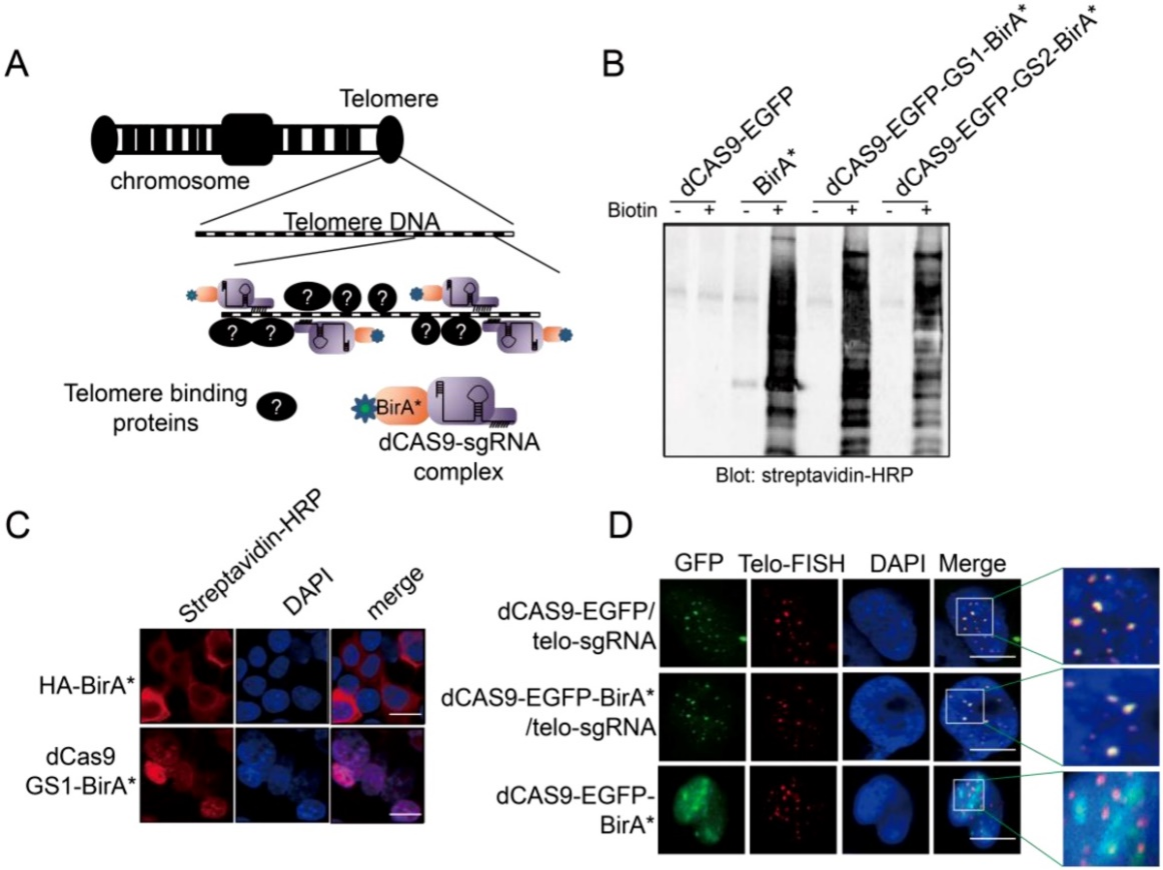
** A.** Diagram to show the design of CASID and its working mechanism. Telomere DNA repeats were selected as a targeted sequence. The sgRNA here represents the sgRNA targeting the telomere DNA repeats (telo-sgRNA). **B.** The whole cell protein extracted from HEK293 cells transfected with BirA, dCAS9-BirA* fusion constructs were detected by Western blot using Streptavidin-HRP. The dCAS9-EGFP transfected cells were used as negative control and dCAS9-BirA* fusion constructs have either GS1 or GS2 linker. **C.** The HEK293 cells transfected with HA-BirA* or dCAS9-GS1-BirA* were detected for biotinylated proteins using Streptavidin-HRP. DAPI indicates the nucleus. The bar indicates the scale of 10 um. **D.** Localization of dCAS9-EGFP and dCAS9-EGFP-BirA* fusion proteins in HEK293 nucleus. HEK293 cells transfected with dCAS9-EGFP serves as negative control. Tel-FISH represents the location of telomeres (red). DAPI indicates the nucleus. The enlarged nuclear area is enriched with telomere FISH signal (red) and dCAS9-EGFP signal (green). The yellow dots represent the overlapping between the telomere FISH signal and dCAS9-EGFP signal.

To capture the proteins that bind to a specific gene locus, we took advantage of the CRISPR technology and fused BirA* with dCAS9 to establish the targeting tool CASID (**Fig 1A**)^30^. In order to conveniently monitoring the location of the fused protein, EGFP coding sequence was also inserted between dCAS9 and BirA*. Moreover, to ensure the re-constructed protein enter nucleus, an NLS (nuclear localization signal) was included in the construct. To prevent the possible functional intervention between dCAS9 and BirA* when they are expressed in fusion, we screened two rigid linkers with the sequence of EAAAK for 2 repeats (L1) or 3 repeats (L2), and two flexible linkers with the sequence of GGGGS for 2 repeats (GS1) or 3 repeats (GS2). We found that the enzymatic activity of BirA* was intact in the constructs containing the flexible linkers GS1 and GS2 rather than in the constructs containing the rigid linkers L1 and L2 (**Fig 1B and Supp. Fig 1**) 41. To further confirm that the dCAS9-NLS-EGFP-GS1-BirA* fused protein could enter nucleus and BirA* still maintains its enzymatic activity, the construct was transfected into HEK293 cells. By detecting the EGFP, we confirmed that the fused protein could enter the nucleus without obvious leakage in cytosol no matter the construct was highly expressed or mildly expressed (**Supp. Fig 2**). As shown in **Fig 1C**, the biotinylated proteins were easily observed in the nuclei of the HEK293 cells transfected with dCAS9-NLS-EGFP-GS1-BirA*, suggesting BirA*, as well as the NLS (Nuclear Localization Signal) inserted to the construct, worked properly. Then the functional integrity of dCAS9 was evaluated based on the observation of EGFP signal in the nucleus with the presence of small guiding RNA (telo-sgRNA) targeting telomere in HEK293 cells. Both dCAS9-EGFP and dCas9-EGFP-BirA* were observed as multiple spots in nuclei. As expected, these EGFP spots co-localized with the telomeres as revealed by telomere-specific DNA FISH (**Fig 1D**). When telo-sgRNA was absent, the dCAS9-EGFP-BirA* did not show co-localization with telomere. These data indicated that dCas9-EGFP-BirA* can be targeted to the telomere DNA by telo-sgRNA. Together, these results supported that the dCAS9-NLS-EGFP-GS1-BirA* construct is functional and can be used for targeted protein modification in a gene locus-specific way.

### Capturing of telomere binding proteins

**Figure 2:**
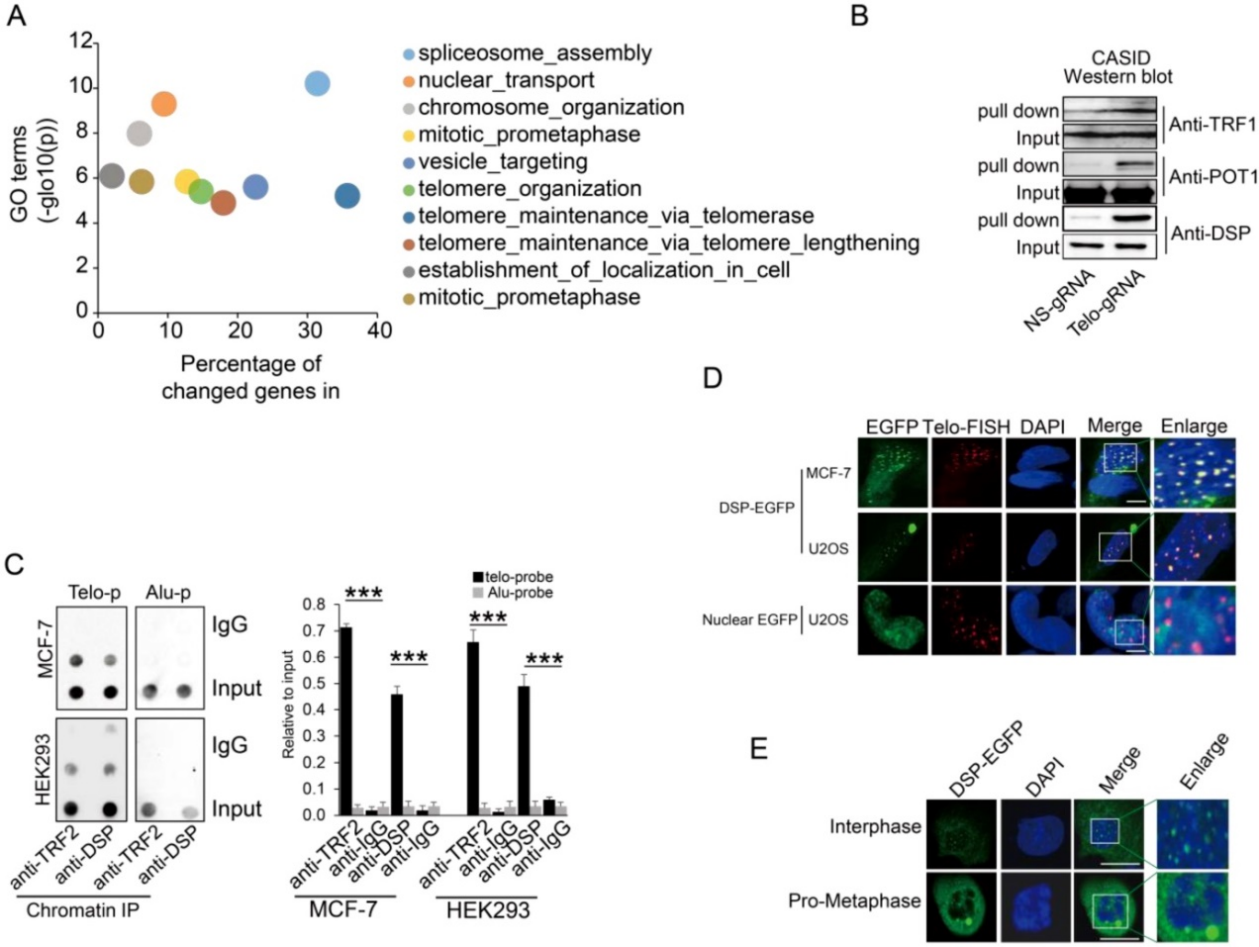
** A.** Gene ontology analysis of proteins identified by CASID targeting telomeres. **B.** Western blot detection of TRF1, POT1, and DSP in the proteins pulled down by CASID with the presence of telo-sgRNA in HEK293 cells. Input represents the initial nuclear extract before the pull-down mediated by CASID. **C.** left, ChIP assay of telomere binding of TRF2 or DSP in MCF-7 cells and HEK293 cells. The chromatin DNA precipitated by anti-DSP and anti-TRF2 antibodies were analyzed by dot blot using the probes recognizing telomere (Telo-p) or Alu DNA repeats (Alu-p). The detection of Alu DNA repeats serves as negative control. The dot-blot was performed at least three times and the represented one is shown. Right, the histogram shows the quantitated results of the dot blots. The triple asterisks (***) indicates a p-value less than 0.01. **D.** Fluorescence imaging of DSP-EGFP in MCF-7 cells and U2OS cells. The EGFP signal indicates the localization of DSP-EGFP (green) and the Telo-FISH signal indicates the localization of telomeres (red). The overlapping between DSP-EGFP and Telo-FISH is in yellow as revealed in the enlarged nuclear area. The dCAS9-EGFP construct without telo-sgRNA was used for U2OS transfection and the imaging of EGFP serves as negative control. **E.** DSP localization in interphase and pro-metaphase nucleus. DSP-EGFP indicates the DSP locations and DAPI indicates the nucleus.

Given that the localization of the fused protein CAS9-NLS-EGFP-GS1-BirA* to the telomere was as expected, the CASID was performed with or without the presence of telo-gRNA in HEK293 cells. The biotinylated proteins were pulled down through the streptavidin beads and further analyzed by LC-Mass. To reduce the potential contamination by the cytoplasmic proteins, only the nuclear proteins extracted from HEK293 cells were used for the streptavidin-mediated pull-down. The comparison was conducted between the samples with telo-gRNA and without telo-gRNA. Across the four independent replicates, we identified 281 unique proteins (-log (P)>150) that were commonly identified in all four replicates and were among the top 25% of total proteins presented only in the samples with telo-gRNA. We also identified another set of 86 unique proteins that were among the top 10% of total proteins showing much higher scores in the samples with telo-gRNA than in the samples without telo-gRNA (differential -log(P)>100), that is consistent in all the replicates. Gene ontology analysis of the coding genes of these total top 367 proteins confirmed that many of these genes are involved in telomere maintenance or elongation (**Fig 2A**). The genes that were enriched in the telomere-related GO terms include TERF2, along with other known telomere binding proteins such as DKC1, TERF2IP, and TNKS1BP1 etc.^42^, indicating CASID is a reliable method to identify the telomere-specific binding proteins. We further compared the telomere-associated proteins identified by different approaches such as QTIP 36 and PICH 37, to our gene list. Surprisingly, only four genes including TRF2, TRF2IP, SNW1, and WDR82 were found by all three technologies (**Supp Fig 3**). Intriguingly, SNW1 and WDR82 were not previously known to be telomere-associated proteins, although the currently available data cannot exclude these proteins as telomere binding proteins. We further checked the CASID data targeting the rDNA repetitive genomic region (data not shown) and found no overlapping with the CASID data targeting telomere, suggesting the gRNA is capable of determining the target specificity of CASID.

Since many previously known telomere-binding proteins such as the other shelterin proteins, were missing in our CASID pull-down the list, we wondered if they could be detected by Western blotting that is more sensitive than LC-MS. In accordance with our expectation, the representative shelterin proteins TRF1 and POT1 were detected in proteins pull-down by CASID with telo-sgRNA (**Fig 2B**), which suggested that the failure of capturing the other shelterin proteins in our CASID analysis is probably because of the low abundance of these proteins in the pull-down protein pool and only antibody-based approaches have the required sensitivity to recognize them.

Next, we selected a top listed protein DSP as a candidate for further validation because DSP is previously known as membrane-associated protein without nuclear function. We successfully validated that DSP associated with telomere by western blot (**Fig 2B**). In support of our observation, a recent study also demonstrated that DSP was co-precipitated by TERB1 (telomere repeat binding protein 1) in mouse testis cells 43. Since this was the first time that DSP was identified potentially working as a nuclear protein, we attempted to pull down DSP associated proteins in the nucleus. Gene ontology analysis of the DSP associated proteins solely in the nucleus identified by LC-Mass exhibited mainly the nuclear matrix related GO terms including the telomere capping complex (**Supp Fig 4**). To further confirm that DSP could bind to telomere in the nucleus, the ChIP assay was performed. As shown in **Fig 2C**, by pulling down DSP or TRF2, the telomere DNA, but not the Alu repeat DNA, was co-precipitated in both MCF-7 and HEK293 cells, suggesting both DSP and TRF2 bind to telomere DNA. Next, we ectopically expressed DSP-EGFP fusion protein in both MCF-7 cells and U2OS cells. In addition, the telomere DNA FISH was applied to indicate the locations of interphase telomeres that were observed as multiple spots within the nucleus (**Fig 2D**). As expected, the EGFP signal from the DSP-EGFP fused protein was found to be shown as multiple spots in the nucleus and overlapped with the telomere FISH signal (**Fig 2D**). As the negative control, the nuclear localized EGFP had no spots-like localization and did not overlap with the telomere FISH signal (**Fig 2D**). Interestingly, we only observed DSP signal at telomeres in interphase nucleus but not in the pro-metaphase nucleus, suggesting the binding of DSP to telomere is cell cycle-dependent (**Fig 2E**).

### C-terminal domain is required for DSP binding to telomere

**Figure 3:**
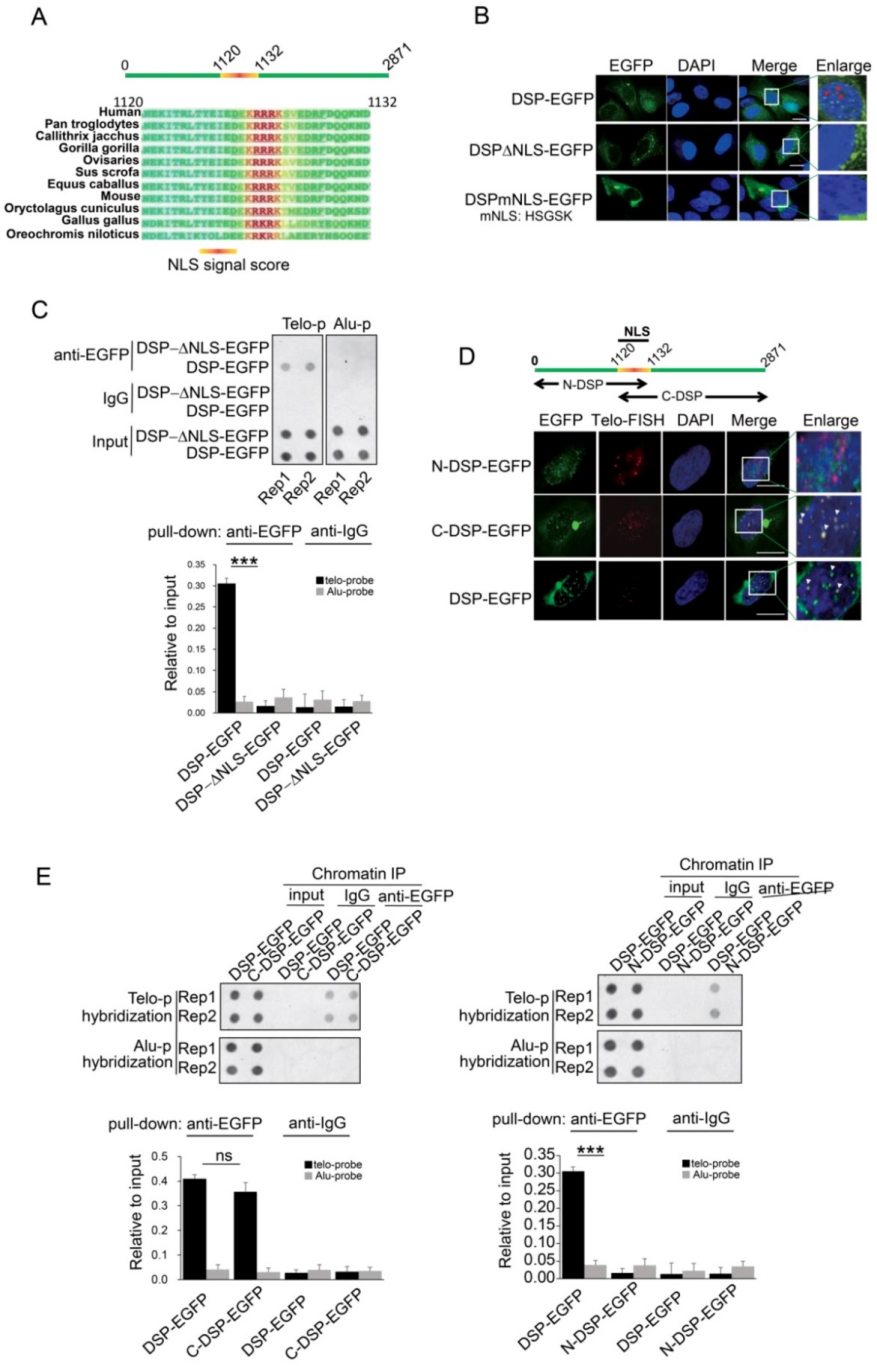
** A.** Prediction of NLS sequence in DSP using Nucpred algorism is shown at the top. The conservation of the NLS of DSP across many species is shown at the below. **B.** Fluorescence imaging of the nuclear localization of DSP-EGFP fusion protein and the cytoplasmic only localization of DSP^△NLS^-EGFP and DSPmNLS-EGFP. The red arrows indicate the green dots formed by DSP-EGFP in nuclei. **C.** Top, ChIP assay using anti-EGFP antibody in the HEK293 cells transfected with either DSP-EGFP fusion protein or DSP^△NLS^-EGFP mutant. Alu DNA repeats serve as negative control. The dot-blot was performed at least three times and the represented one is shown. Bottom, the histogram shows the quantitated results of the dot blots. The triple asterisks (***) indicates a p-value less than 0.01. **D.** Fluorescence imaging of N-DSP-EGFP and C-DSP-EGFP fusion proteins (green) in HEK293 cells. The overlapping between EGFP (green) and Telo-FISH (red) is in yellow as revealed in the enlarged nuclear area (white arrows). DAPI indicates the nucleus. **E.** Top, ChIP assay using anti-EGFP antibody in cells transfected with C-DSP-EGFP (left) or N-DSP-EGFP (right). Alu DNA repeats serve as negative control. The dot-blot was performed at least three times and the represented one is shown. Bottom, the histograms show the quantitated results of the dot blots. The triple asterisks (***) indicates a p-value less than 0.01 and ns. indicates the insignificant difference.

Considering DSP is able to enter the nucleus, we speculated that an NLS motif might exist in DSP protein. Then we performed the NucPred analysis 44, a strong NLS motif was predicted in DSP protein and this motif is conserved in many species (**Fig 3A**). To determine whether this NLS is essential for mediating the nuclear localization of DSP, two mutant DSPs with NLS deletion (DSP**^△NLS^**-EGFP) or NLS mutation (DSPmNLS-EGFP: KRRRK to HSGSK) were constructed. Consistent with our prediction, both DSP**^△NLS^**-EGFP and DSPmNLS-EGFP failed to enter the nucleus (**Fig 3B**). Moreover, the DSP**^△NLS^**-EGFP failed to co-precipitate the telomere DNA after being transfected into HEK293 cells in the anti-EGFP ChIP assay (**Fig 3C**), suggesting the predicted NLS motif is essential for the nuclear localization and telomere binding of DSP.

DSP is a large protein containing 2871 amino acids. Previous studies indicated that DSP forms homodimer containing two globular end domains and a central rod domain. The N-terminal domain of DSP is essential to bind to other desmosome proteins such as PLAK, PG etc. and the C-terminal domain interacts with filamentous proteins 45. To examine which part of DSP is required for its binding to the telomere, the DSP was split into N-terminal and C-terminal fragments with both containing the NLS and being fused with EGFP (**Fig 3D**). Transfection of these constructs into HEK293 cells disclosed that N-DSP-EGFP localized in the nucleus as blurred spots without overlapping with the telomere FISH signal (**Fig 3D**). However, the C-DSP-EGFP signal showed very similar distribution pattern as the full DSP-EGFP showed in the nucleus. Both the DSP-EGFP and C-DSP-EGFP signal overlapped with the FISH signal of telomere (**Fig 3D, red arrow**), suggesting the C-terminal domain, which is enriched with plakin repeat domain and glycine-serine-arginine rich domain (GSR), was responsible for the telomere binding of DSP. Consistently, the ChIP assay further confirmed that only the C-DSP-EGFP co-precipitated telomere DNA but not the N-DSP-EGFP (**Fig 3E**).

### TRF2 partially mediates DSP binding to telomere

**Figure 4:**
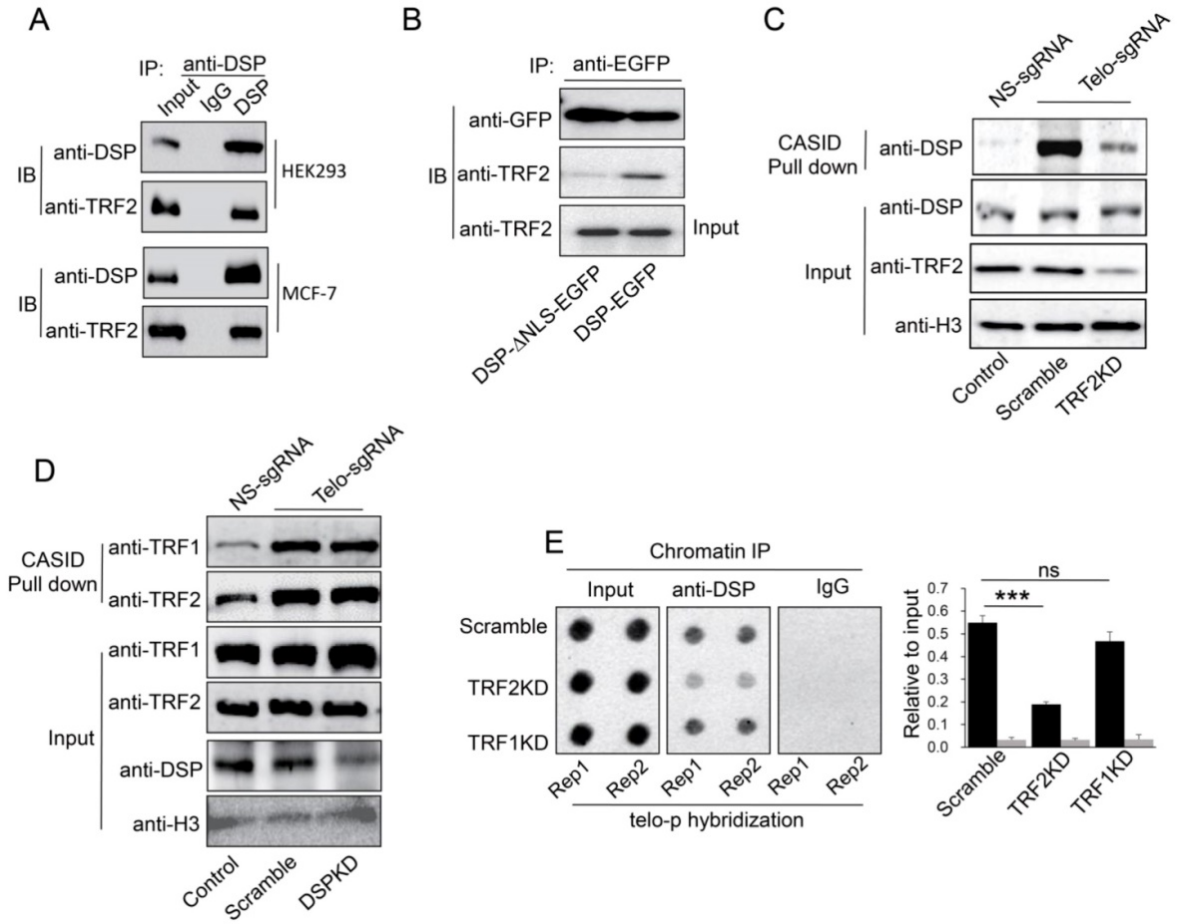
** A.** Co-IP analysis of the interaction between DSP and TRF2 in HEK293 cells and MCF-7 cells. **B.** Co-IP analysis of the interaction between DSP and TRF2 in DSP-EGFP transfected HEK293 cells or DSP^△NLS^-EGFP transfected HEK293 cells. **C.** DSP pull down by telomere targeting CASID with the condition of TRF2 knockdown in HEK293 cells. Histone H3 is the loading control. **D.**TRF1 and TRF2 pull-down assay by telomere targeting CASID with the condition of DSP knockdown in HEK293 cells. **E.** Left, Dot blot of telomere DNA repeats in chromatin pull down by DSP with the condition of TRF1 or TRF2 knockdown. The dot-blot was performed at least three times and the represented one is shown. Right, the histogram shows the quantitated results of the dot blots. The triple asterisks (***) indicates a p-value less than 0.01. ns. indicates the insignificance difference.

If DSP is a telomere binding protein, we wondered whether DSP could interact with shelterin proteins in the interphase nucleus. As shown in **Fig 4A**, we confirmed that TRF2 interacts with DSP in both HEK293 and MCF-7 cells using the co-immunoprecipitation approach. In support of this conclusion, knockdown of DSP abolished the interaction between DSP and TRF2 in the co-immunoprecipitation assay (**Supp. Fig s5**). However, TRF1 did not interact with DSP in both cell lines using the same approach (**Supp. Fig s6**). Moreover, the interaction between TRF2 and DSP was only found when DSP contains the NLS (**Fig 4B**), suggesting the interaction is limited to the nucleus. To investigate whether telomere binding of DSP is TRF2 dependent, we further employed CASID to pull down the telomere proteins with the condition of TRF2 knockdown. As shown in **Fig 4C and Supp. Fig s7**, knockdown of TRF2 significantly reduced the telomere binding of DSP but knockdown of TRF1 did not influence the telomere binding of DSP. Interestingly, when DSP was knocked down, the binding of both TRF1 and TRF2 to telomere did not change obviously (**Fig 4D**), suggesting telomere association of shelterin complex is independent of DSP but TRF2 is required for the telomere binding of DSP. Consistently, ChIP assay also demonstrated that TRF2 knockdown, but not TRF1 knockdown, resulted in the reduction of the co-precipitated telomere DNA by the anti-DSP antibody (**Fig 4E**).

### DSP binding to telomere is telomerase-dependent

**Figure 5:**
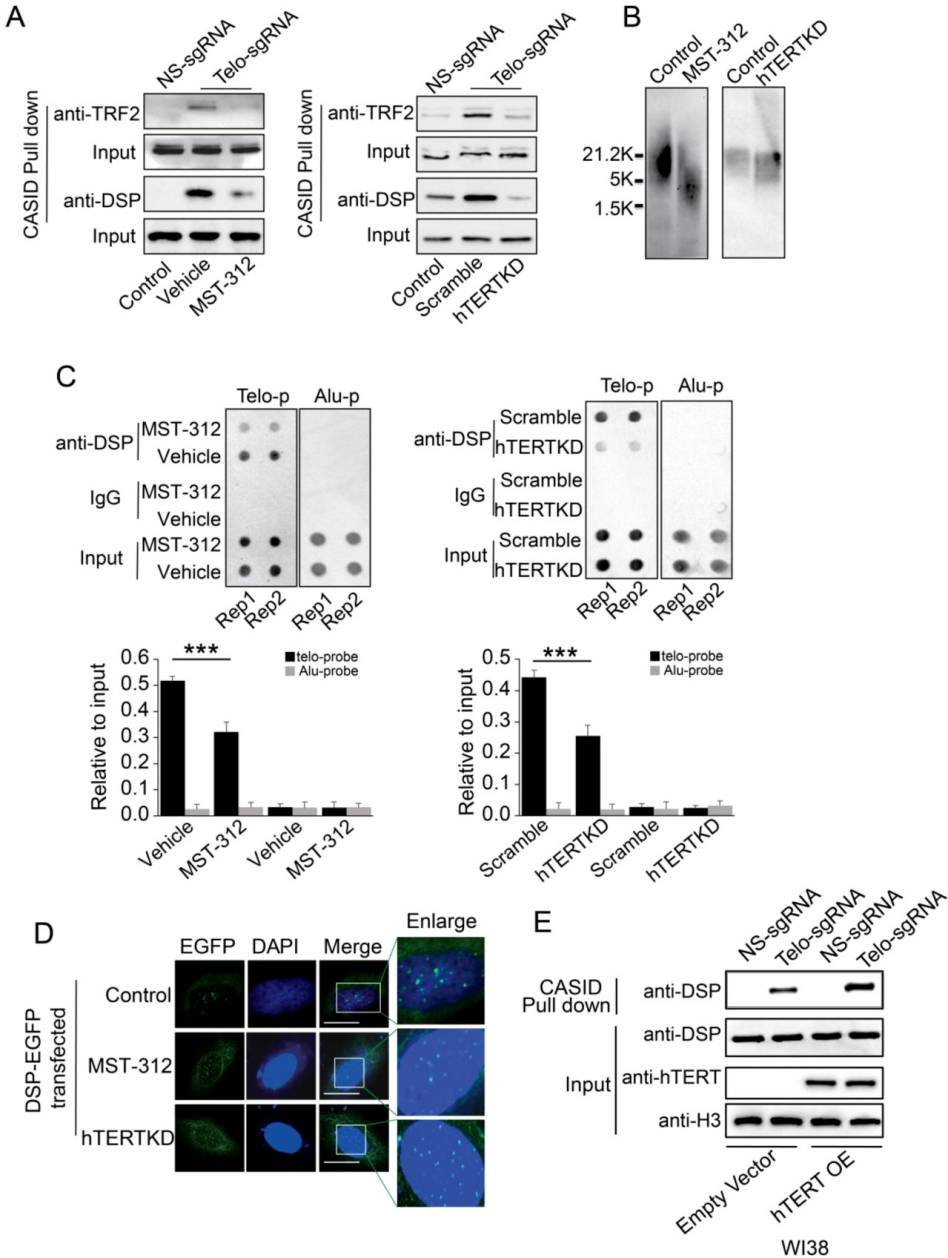
** A.** TRF2 or DSP1 pull down by telomere targeting CASID with the condition of MST-312 treatment (left) or hTERT knockdown (right) in MDA-MB-231cells. **B.** Southern blot of telomere DNA from MDA-MB-231cells treated with MST-312 or with hTERT knockdown. **C.** Top, Dot blot of telomere DNA repeats from the chromatin pull down by DSP from the cells treated by MST-312 (left) or hTERT knockdown (right). Alu DNA repeats serve as negative control. Bottom, the histogram shows the quantitated results of the dot blots. The triple asterisks (***) indicates a p-value less than 0.01. **D.** Fluorescence image of DSP-EGFP transfected MDA-MB-231 cells with the conditions of MST-312 treatment or hTERT knockdown. **E.** Pull down assay of DSP by CASID targeting telomere in WI38 cells with or without ectopically expression of hTERT gene.

In cancer cells, active telomerase is required to maintain the telomere. To investigate whether the telomere binding of DSP is telomerase-dependent, MST-312, an EGCG analogous compound with telomerase inhibitory activity, was applied to treat MDA-MB-231 cells that are more sensitive to MST-312 by comparing to other cell lines 46. MST-312 treatment for 5 days significantly reduced the telomere binding of TRF2 and DSP according to the quantitation of proteins pulled down by CASID with the presence of telo-sgRNA (**Fig 5A**). Consistently, knockdown of hTERT in MDA-MB-231 cells also reduced the telomere binding of TRF2 and DSP (**Fig 5A, Suppl. Fig s8**). Since MST-312 acts as an inhibitor of the enzymatic activity of hTERT, we hypothesized that the shortening of telomere DNA length could cause the reduced telomere binding of both DSP and TRF2 upon treatment by MST-312 and hTERT knockdown. We applied southern blot to measure the length of telomere DNA after the MDA-MB-231 cells were treated by MST-312 or the hTERT was knocked down. As shown in **Fig 5B**, both MST-312 stimulation and hTERT knockdown decreased telomere DNA length significantly. To further demonstrate that telomere binding of DSP is telomerase activity-dependent, ChIP assay was performed. The ability of DSP to precipitate telomere DNA was found to be reduced when the cells were treated by MST-312 or the cells had hTERT knockdown (**Fig 5C**). Furthermore, we found that the fluorescence signal of DSP-EGFP still showed as multi spots within the nucleus after the cells were treated by MST-312 or the cells had hTERT knockdown, although the signal becomes weaker. We speculated that the MST-312 treatment and hTERT knockdown only mildly trim down the length of telomere DNA in MDA-MB-231 cells (**Fig 5D**). Finally, we tested if DSP binding to telomere is partially hTERT dependent in lung fibroblast cell WI38. WI38 cells do not have hTERT expression and we ectopically expressed hTERT. As shown in **Fig 5E**, hTERT expression in WI38 cells slightly increased DSP binding to telomere as revealed by the CASID mediated pull-down assay. Together, these observations suggest that DSP binding to telomere is partially influenced by hTERT. Since hTERT expression will extend the length of the telomere, we suspected that the binding of DSP to telomere is indirectly influenced by hTERT.

### Loss of DSP induces DNA damage response

**Figure 6:**
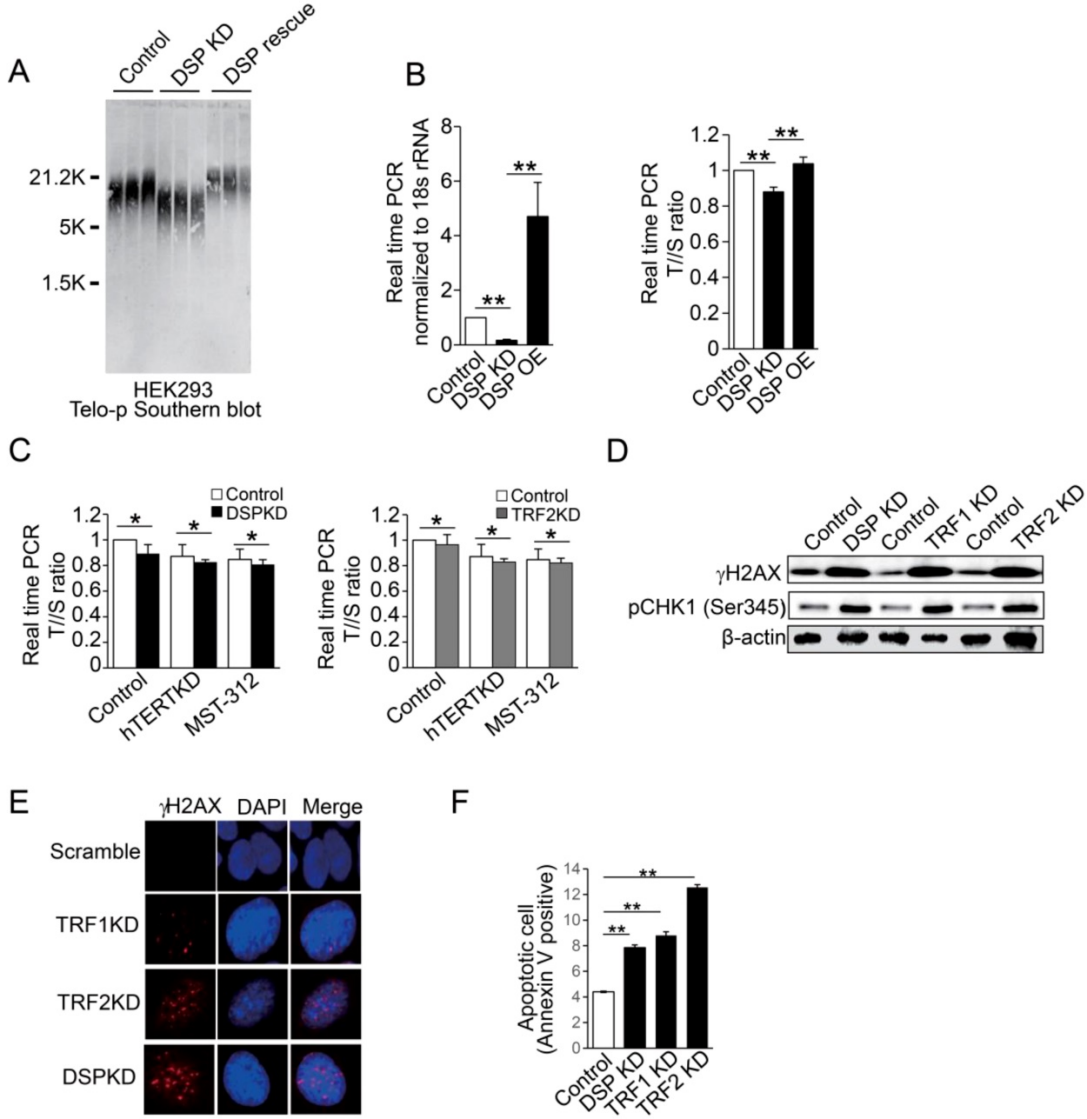
** A.** Southern blot of telomere DNA in three independent HEK293 cell clones for each condition including the HEK293 cells, HEK293 cells with DSP knockdown, and HEK293 cells with DSP knockdown but with the rescue of DSP. **B.** Copy number measurements of telomere DNA repeats by normalizing to the single copy genome sequence. Left, DSP expression upon DSP knockdown or DSP overexpression in HEK293 cells. Right, the telomere to single copy genome sequence ratio (T/S). **C.** Copy number measurements of telomere DNA repeats by normalizing to the single copy genome sequence, with or without DSP knockdown (left) or TRF2 knockdown (right) in combination with hTERT knockdown or hTERT inhibition by MST-312. **D.** Western blot of phosphorylated γH2AX and phosphorylated CHK1 (pCHK1 (Ser345)) in HEK293 cells with DSP knockdown, TRF1 knockdown, or TRF2 knockdown. **E.** Immunofluorescence of phosphorylated γH2AX in HEK293 cells with TRF1, TRF2, and DSP knockdown respectively. **F.** Apoptotic cell measurement by flow cytometry in cells with DSP, TRF1, or TRF2 knockdown respectively. The errors represent the SD of the mean from at least 3 independent experiments (B. C. F.); *, p<0.05;**,p<0.01.

Finally, we examined the biological importance of DSP in the nucleus. Interestingly, depletion of DSP leads to obvious shortening of telomere DNA length as indicated by Southern blot in several different cell lines (**Supp. Fig s9**). The direct correlation between DSP and the length of telomere was further confirmed when DSP was rescued in HEK293 cells with DSP knockdown (**Fig 6A and Supp. Fig s10**). We also applied real-time PCR to evaluate the change of telomere DNA length upon DSP knockdown or overexpression in HEK293 cells. The consistent shortening or extension of telomere DNA by DSP knockdown or overexpression was also validated (**Fig 6B**). Next, we further found that DSP knockdown sensitizes the cells to hTERT knockdown or inhibition by MST-312. Such effect is very similar to the condition of TRF2 knockdown (**Fig 6C**), suggesting DSP, just like TRF2, has non-overlapped function with hTERT in protecting the telomere. To assess whether DSP plays an important role in protecting the telomere from damage just like shelterin proteins, the γH2AX and phospho-CHK1 (Ser 345) were used to demonstrate the cell response to DSP knockdown in HEK293 cells. DSP knockdown increased γH2AX and phospho-Chk1 (Ser 345) that was comparable to the change caused by TRF1 or TRF2 knockdown (**Fig 6D**). DSP, TRF1 or TRF2 knockdown resulted in the increasing of γH2AX (**Fig 6E**), suggesting DSP is essential for preventing telomere DNA damage. Since increased DNA damage may induce the cell death, flow cytometry analysis of apoptotic cells was performed and as expected, DSP knockdown significantly increased the cell apoptosis which was almost equivalent to the effect of TRF1 knockdown (**Fig 6F and Supp. Fig s11**). Consistent with a previous study 47, TRF2 knockdown induced the most significant cell apoptosis (**Fig 6F**). Together, our findings indicated that DSP is a telomere binding protein and contributes to the maintenance of telomere DNA intact and protection from DNA damage.

## Discussion

It was a completely unexpected finding that DSP is one of the top hits of telomere-associated proteins in the CASID analysis. DSP is previously known as a cytoskeleton protein mediating the anchorage of cytoskeleton filamentous fiber to the desmosome 48. In a recent study, DSP expression was found to be influenced by the telomere length owing to the “position effect” 49. However, this study did not explore if DSP influences telomere length as feedback. The appearance of the desmosome proteins in the nucleus indicated that the nuclear skeleton, or better known as nuclear matrix, shares some common mechanisms with the cytoskeleton. Previous studies already indicated that the nuclear lamina, a proteinaceous meshwork located on the inner side of the nuclear membrane, provides the attachment sites for chromatin 50. If DSP binds to the telomere, an interesting proposition is that they may mediate the attachment of telomere to the inner membrane lamina or the intermediate filament. Eventually, the telomeres may attach to the nuclear membrane via these nuclear matrix proteins. Published studies indicate that the telomere positioning in the nucleus is lamin-dependent 51-53. Interruption of the interphase positioning of telomere may result in telomere damage, which is consistent with what we observed. However, the detailed mechanism in telomere positioning and how DSP contributes to this mechanism are out of the scope of this study and a future prospective study is ensured to understand this critical event in interphase telomere localization.

Although depletion of DSP induced both DNA damage response and cell apoptosis, it is not conclusive that DNA damage response is the only reason of increased cell apoptosis. Previous studies have demonstrated that DSP recruits Ninein, Lis1, and Ndel1, to cell cortex upon epidermal differentiation 54. These proteins are centrosome proteins required for microtubule anchoring. Loss of DSP prevents accumulation of cortical microtubules in vivo and in vitro 55. Because of the critical role of the centrosomes during cell division, loss of DSP may block the normal function of centrosomes and induce the cell death independent of DNA damage. However, our observation of telomere binding of DSP and its dissociation from M-phase chromosomes imply that there might be functional connections between the DSP binding of telomere and DSP mediated centrosome dynamics.

Some other studies also applied different technologies to capture the telomere-associated proteins 36, 56. However, none of these studies identified DSP as telomere-associated protein. To observe how much agreement these different technologies commonly have, we compared the telomere-associated protein lists generated by PICh, QTIP 36 and CASID. Surprisingly, the extent of overlapping among the telomere-associated protein lists was similar and insignificant. Only less than 6% of overlap can be observed between any two of these lists. However, when we compared the protein lists generated by previously published CASID and our list, there are 11.2% (62 overlapping proteins between 258 and 359 targets) proteins in common. Thus, it is quite likely that the technologies may be the reason for the inconsistency because the data generated by the two CASID studies have better consistency. Nevertheless, the overlapping of 11.2% between the two CASID is still far from satisfying. Our CASID performed much better than the previously published CASID targeting telomere because we generated a list of novel telomere binding proteins over 300. Even though many of these targets are probably noises, the successful validation of DSP, one of the top hits in our CASID analysis, suggest that our list contains some novel telomere binding protein. The previous CASID study also detected DSP in their list but it was not further considered as an important novel finding because DSP didn't survive the cut-off threshold 30. The major difference between our CASID approach and the published one is the design of linkers. Since only the rigid GS linkers, but not the flexible linkers, maintained the activity of BirA* in the dCAS9-BirA* fused protein, we suspect that the inclusion of the GS linker in our approach might improve the sensitivity and accuracy of the CASID. The alternative explanation might be the quality of the LC-MS analysis. Since there are various LC-Mass equipment and technologies, the deviations generated by LC-MS can be crucial. In support of this supposition, all these studies considered the shelterin proteins as positive control but none of them was able to identify all the known shelterin components but only a few of them without consistency. Thus, applying standard LC-MS procedure and using the same LC-Mass equipment might be helpful to solve the problem of data inconsistency in the future.

## Supplementary Material

Supplementary figures and tables.
