# Supplementary material for: Nuclear localization of Desmoplakin and its involvement in telomere maintenance
Source: Int J Biol Sci. 2019 Aug 24;15(11):2350–62. doi: 10.7150/ijbs.34450 (PMC6775319; doi:10.7150/ijbs.34450)
Supplement: Supplementary file 1 — Supplementary figures and tables. [file ijbsv15p2350s1.pdf]

Nuclear localization of Desmoplakin and its involvement in telomere maintenance

Peipei Li[1], Yuan Meng[1], , Yuan Wang[1], Jingjing Li[1], Manting Lam[2], Li Wang[1,2]*, Li-jun Di[1]*

[1]*Cancer Center, Faculty of Health Sciences, University of Macau, Macau, SAR of China.*

[2]*Metabolomics and proteomics Core, Faculty of Health Sciences, University of Macau, Macau, SAR of China.*

* Correspondence authors

Mailing address: Room 4009, Faculty of Health Sciences(E12),

University of Macau, Avenida da Universidade, Taipa, Macau, China

Tel. 853-88224497

Fax. 853-88222314

Email:lijundi@umac.mo

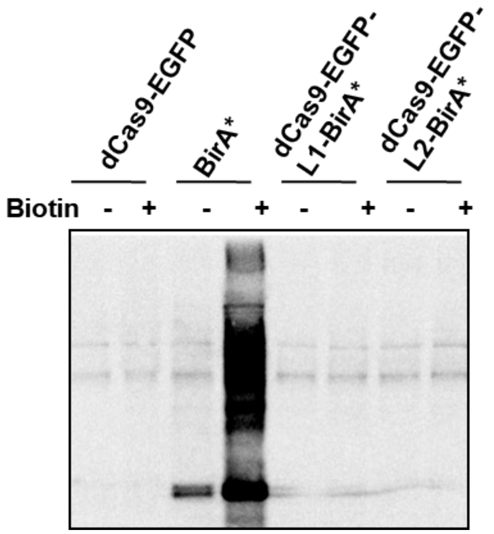

Supp Fig1. Detection of proteins extracted from HEK293 cells transfected with dCas9-EGFP, BirA* or dCAS9-BirA* fusion constructs using streptividin-HRP. The western blot of proteins extracted from HEK293 cells transfected with dCAS9-EGFP served as the negative control and the western blot of proteins extracted from HEK293 cells transfected with BirA* served as positive control. The dCAS9-BirA* fusion constructs contain either L1 or L2 rigid linker.

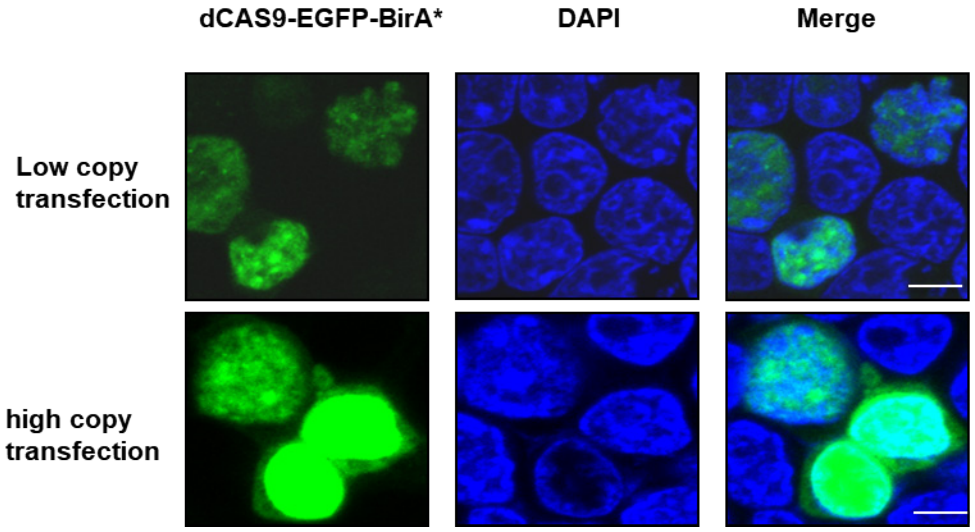

Supp Fig2. Localization of dCAS9-EGFP-BirA* in HEK293 cell nucleus. HEK293 cells were transfected with dCAS9-EGFP-BirA* using either high quantity of vector or low quantity of vector with all the other transfection conditions the same. The images of the live cells were captured using the Confocal fluorescence microscope.

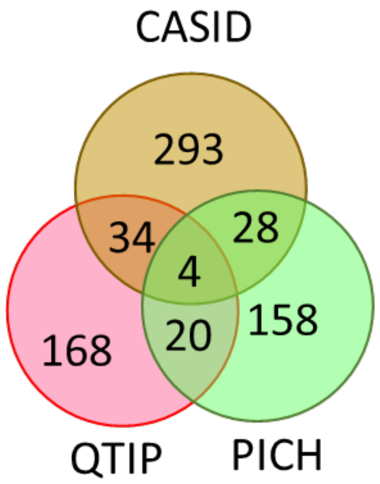

Supp. Fig3 The venn diagram to show the overlapping of telomere associated proteins pulled down by 3 different technologies including CASID, QTIP and PICH.

GO term	Total Gene	Changed G	Score	p-value
GO:0005635_nuclear_envelope	256	19	8.003731	-11.544
GO:0034399_nuclear_periphery	73	11	16.24982	-10.1783
GO:0016363_nuclear_matrix	65	10	16.59073	-9.39089
GO:0046930_pore_complex	86	11	13.79346	-9.3858
GO:0031965_nuclear_membrane	150	13	9.346111	-8.85704
GO:0005637_nuclear_inner_membrane	29	6	22.31167	-6.6384
GO:0005819_spindle	178	10	6.058413	-5.19933
GO:0030117_membrane_coat	66	6	9.803613	-4.47949
GO:0030120_vesicle_coat	42	5	12.83807	-4.3823
GO:0030126_COPI_vesicle_coat	12	3	26.95994	-3.79095
GO:0000782_telomere_cap_complex	12	2	17.97329	-2.27528

Supp. Fig4 The GO analysis of DSP interacting proteins in nucleus.

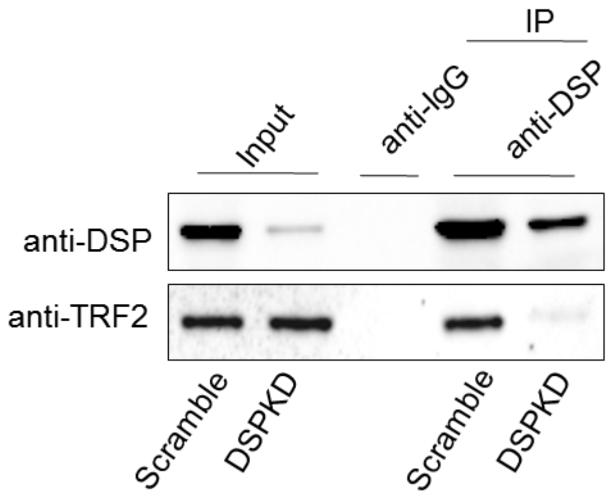

Supp Fig5. Co-IP analysis of interaction between DSP and TRF2 with the condition of DSP knockdown. The anti-DSP antibody was applied to pull down proteins from HEK293 cells and anti-DSP and anti-TRF2 antibodies were applied to detect the target proteins from the pull down proteins. anti-IGG serves as negative control.

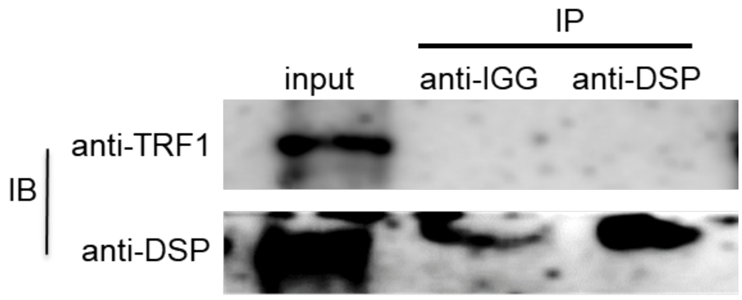

Supp Fig6. Co-IP analysis of interaction between DSP and TRF1. The anti-DSP antibody was applied to pull down proteins from HEK293 cells and anti-DSP and anti-TRF1 antibodies were applied to detect the target proteins from the pull down proteins. Anti-IGG serves as negative control.

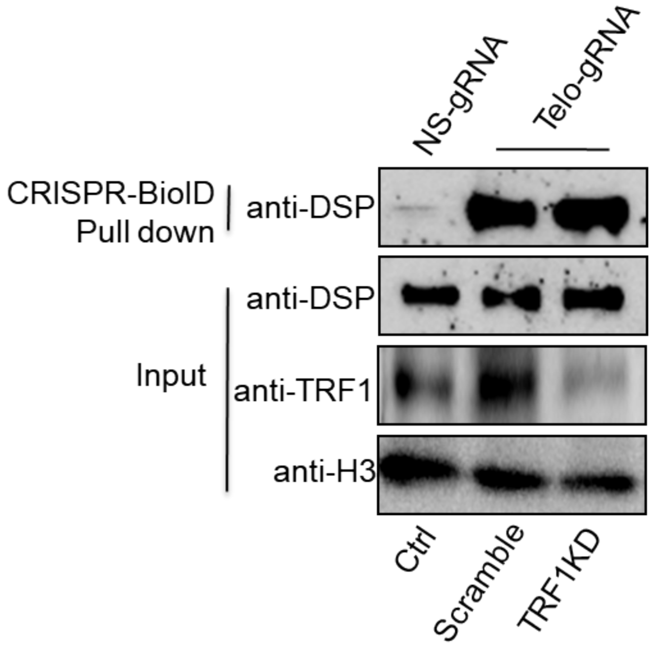

Supp Fig7. Telomere targeting CASID pull down was performed with or without the condition of TRF1 knockdown in HEK293 cells. The DSP was detected in the pull down proteins. The scramble represents the negative control for TRF1 knockdown and the NS-gRNA represent the CASID pull down with presence of none targeting sgRNA.

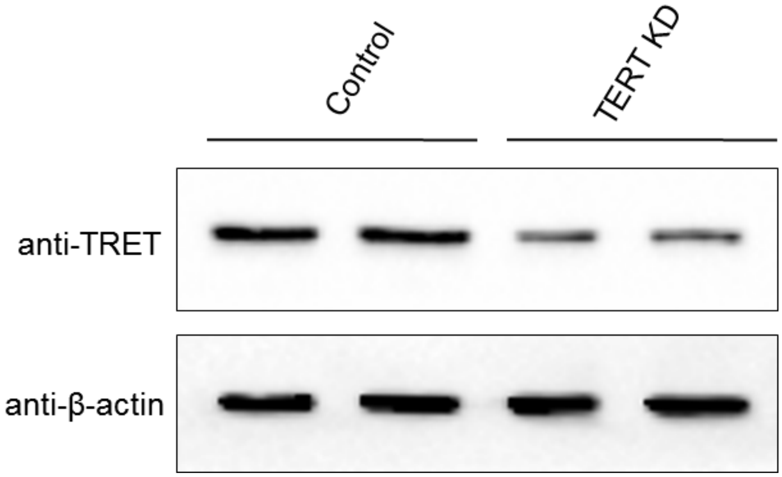

Supp Fig8. Validation of hTERT knockdown in HEK293 cells.

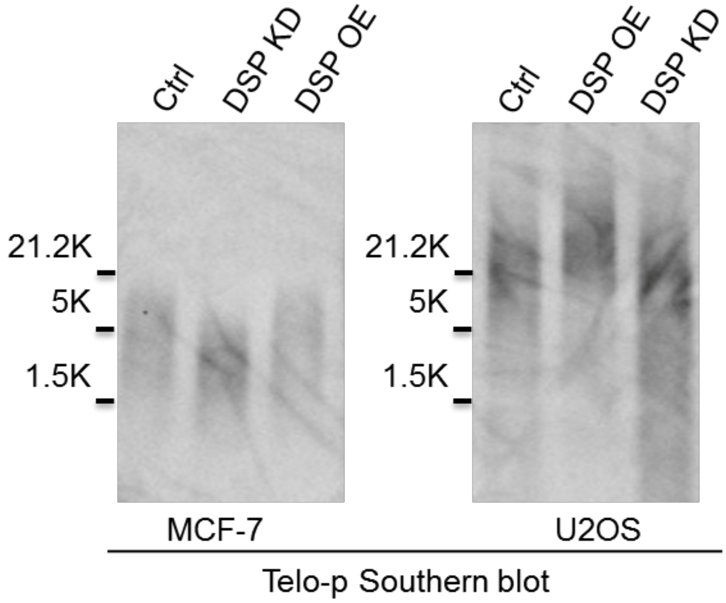

Supp. Fig9 Southern blot analysis of telomere DNA length in MCF-7 cells and U2OS cells. Both cells were treated by DSP knockdown or DSP overexpression. The markers indicate the size of the DNA. The probe for southern blot is targeting telomere.

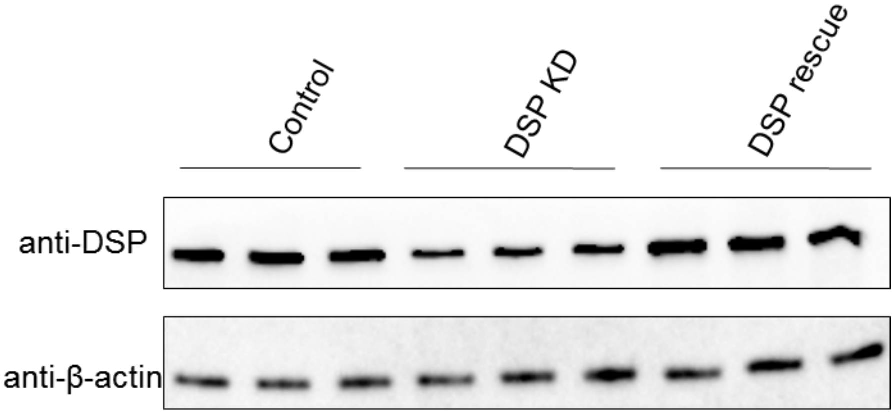

Supp. Fig10 Western blotting of DSP with the condition of DSP knockdown and DSP rescue in HEK293 cells.

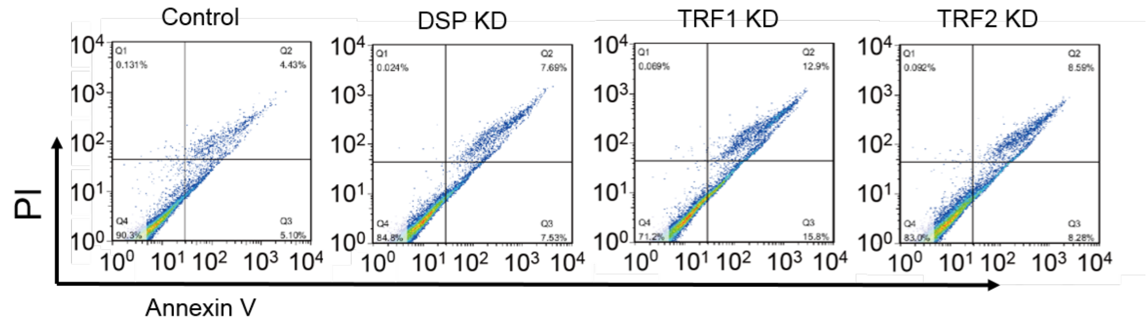

Supp Fig11 Flow cytometry analysis of apoptotic cells by Annexin V and PI staining upon DSP, TRF1 or TRF2 knockdown.

Materials and Methods

Constructsand antibodies

BirA*-dCas9-EGFP construct was generated by subcloning BirA*-HA from pcDNA3.1 MCS-BirA(R118G)-HA (Addgene #36047) into pSLQ1658-dCas9-EGFP (Addgene #51023), BglII and NCOI were selected restriction enzyme digestion sites. SgRNA-Telo construct was created by subcloning telomere repeat sequence into gRNA_Cloning Vector (Addgene #41824) with Gibson Assembly assay. To construct the $DSP^{\Delta NLS}$, N-DSP and C-DSP expression vectors, gene fragments were generated from 1136-Desmoplakin-GFP (Addgene #32227) by PCR, then the PCR fragments were cloned into pLJM1-EGFP. All the antibodies used in this research were as follow: anti-GFP (MBL, M048-3), anti-TRF1 (Santa cruz, sc-56807), anti-POT1 (Santa cruz, sc-81711), anti-TRF2 (Novus Biologicals, NB110-57130), anti-γH2AX(EMD Millipore, 05-636), anti-DSP (Abcam, 118804), anti-Phospho-Chk1 (Ser345) (Cell Signaling technology, #2341). Telo-sgRNA sequence: AGGGTTAGGGTTAGGGTTA

Linker sequences:

GS1：ggtggtggcggttcaggcggaggtggctct

GS2：ggtggtggcggttcaggcggaggtggctctggcggtggcggatcg

L1：gaggccgccgccaaggaggccgccgccaag

L2：gaggccgccgccaaggaggccgccgccaaggaggccgccgccaag

Cell culture and transfection

Human embryonic kidney cell line HEK293, human bone osteosarcoma epithelial cell line U2OS, human breast cancer cell line MDA-MB-231 and MCF-7 cells were cultured in Dulbecco's modified Eagle medium (DMEM, Gibco) with 2mMglutamine (Gibco) in 10% fetal bovine serum FBS (Gibco). All cells were maintained at 37℃ and 5% CO2 in a humidified incubator. For HEK293 cells, plasmid transfections were carried out using PEI, for other cell lines, plasmids were transfected with Lipofectamine 3000 (Thermo Fisher Scientific) according to the manufacturer's instructions.

Rescue of DSP upon DSP knockdown in HEK293 cells.

To rescue the expression of DSP in the DSP depleted cells, DSP plasmid was transfected into HEK293 cells stably expressing shDSP (targeting 3'-UTR) by using PEI, then the cells were selected by G418 for two weeks, finally collect the cells and perform the western blotting and southern blotting.

Detection of biotinylated proteins and BioID pull down

Cells were seeded into a 6-well plate cell culture ready for transfection by the following vectors: empty vector with or without biotin, dCas9-EGFP-BirA* vector with or without biotin), after transfection, 50μM biotin was added into the culture medium 3h post-transfection. 24 h later, the 6-well plate cultured cells were harvested for immunoblot assay, the cells were lysed by 100 μL cell lysis buffer on ice for 30 min with the presence of protease inhibitors, and further sonicated and centrifuged at 15,000g for 10 min at 4°C. These samples were analyzed by SDS-PAGE electrophoresis and protein was detected by streptavidin-HRP (Sigma).

For large-scale ($5x10^7$cells) BioID pull down assay, cells were seeded into two 15-cm dishes for each experimental condition. 50μM biotin was added into the culture medium 3h post-transfection. 48 h later, the cells were lysed and the nuclear proteins were extracted in the presence of protease inhibitors. The supernatant of cell lysates were gently transferred to 2-mL tubes and were diluted to 2.5 fold with pre-chilled 50 mM Tris·Cl, pH 7.4. Subsequently the lysates were aliquoted to 1.5 mL per tube. The magnetic streptavidin beads should be equilibrium in 1:1 lysis buffer and 50mM Tris·Cl, pH 7.4. Using the magnetic separation stand to collect the magnetic beads and remove the supernatant after equilibrium. The nuclear extracts and beads were mixed gently, and they were incubated on the rotator over night at 4°C.The magnetic beads were washed sequentially by Wash Buffer 1to 3 for once and by Buffer 4 for twice (Wash buffer 1: 2% SDS in H_2O; Wash buffer 2: 0.1% deoxycholate, 1% Triton X-100, 500mM NaCl, 1 mM EDTA, and 50 mM Hepes, pH 7.5;Wash buffer 3: 250 mM LiCl, 0.5% NP-40, 0.5% deoxycholate, 1 mM EDTA, and 10 mM Tris, pH 8.0;Wash buffer 4: 50 mM Tris, pH 7.4). The eluted protein was further analyzed by Western blot or LC-mass.

LC-mass analysis

The protein-bound beads after pull-down were washed twice by 50 mM ammonium bicarbonate solution. 200 ul of 50 mM ammonium bicarbonate was used to suspend the beads. 7. 4 uL of 0.5 M Tris (2-carboxyethyl) phosphine (final concentration 10 mM) was added to the suspension and mixed for 30 minutes at 40°C. Then 8.8 uL of 0.5 M iodoacetamide (final concentration 20 mM) was applied to isolated proteins at room temperature in dark for 30 min. MS-grade trypsin (1:20 ratio, about 1 ug) was applied to digest the proteins for 16-18h at 37°C. Next, digested peptides were separated from magnetic beads by centrifugation, and adjusted to contain 0.1% TFA. The peptide solutions were cleaned up using C18 ziptip according to the manufacturer instruction. 3.5 μL of 30% ACN/0.1% TFA was applied to elute the proteins from ziptip by pipetting up and down for 20 times. 18 μL of 0.1% formic acid was added into each sample by pipetting up and down, then transferred to an HPLC vial. Finally, 15 μL of sample was carried out with LC-MS analysis (LC, UltimateTM3000 RSLCnano system (thermos Fisher Scientific); MS, Q-exactive TM quadrupole orbitrap mass spectrometer) and protein ID search using shotgun approach. The mass spectrometric data analysis was conducted by using the Peaks Studio 8.5 build 20171002, the sequence search was analyzed by Swiss-Prot database 2016_02 2016-02-17. Max missed tryptic cleavages was two, and one non-specific cleavage was allowed. Fragment mass tolerance for precursor ions and MS/MS fragments were 10 ppm and 0.05 Da, respectively. FDR of Peptide-Spectrum Matches, Peptide Sequences and Protein is no more than 1.0%.

Immunofluorescence staining Cells were seeded into an 8-well chamber cell culture, and plasmids were transfected into the cells using PEI or Lipofectamine 3000. After 48-72 h post-transfection, the cells were fixed for 10 min in 4% paraformaldehyde at room temperature and were permeated with 0.2% TritonX-100 for 10 min. Then the cells were blocked with 1% BSA for 0.5 h, followed by incubation of primary antibodies in primary antibody dilution buffer for 1 hour. Then the chamber was washed in PBST three times and incubated with secondary antibodies for 30min, secondary antibodies included Alexa 488,Alexa 594and streptavidin-Alexa Fluor (Life Technologies). Cells were counterstained with DAPI, and chamber was mounted by anti-fade mounting medium (Beyotime). Finally, the fluorescence was observed by microscope.

PNA FISH and Immunofluorescence with PNA FISH

For PNA FISH, cells were cultured in a 8-well chamber, the chamber was fixed in methanol:acetic acid (3:1) for 10 min at room temperature, and washed in PBS for three time. Next the chamber was immersed in 4% paraformaldehyde

for 10 min at room temperature. After fixation, the chamber was dehydrated using a series of cold ethanol washes (70%, 80%and 100%) and air dried for 10 min. The 500 nM of labeled PNA probe (TelC-Cy3 probe, Panagene) in 70% formamide, 1% (w/v) blocking reagent and 10 mM Tris, pH 7.2 was added into chamber and DNA was denatured at 80℃ for 3 min. Then the chamber was incubated in the dark at 37℃ for 1h. After hybridization, the chamber was washed with 70% formamide, 10 mM Tris pH 7.2 for 10 min and with TNT (0.05 M Tris/0.15 M NaCl/ 0.05% Tween-20, pH 7.5) for 10 min. Cells were counterstained with DAPI, and chamber was mounted by antifade mounting medium (Beyotime).

For IF-PNA FISH, after the incubation of primary antibody and secondary antibody, the chamber was dehydrated using a series of cold ethanol washes (70%, 80% and 100%) and air dried for 10 min. Followed with other FISH steps and image analysis.

Optical Setup and Image Acquisition

For immunofluorescence staining, images were acquired on a Zeiss 710 laser scanning confocal microscope with PLAN-Apochromat 40×/1.40 oil DIC M27 objective or Olympus BX53 microscope with Up lan FLN 40×/0.75 Ph1 objective. For the localization of DSP-EGFP and N/C-DSP-EGFP, images were acquired on a Zeiss 710 laser scanning confocal microscope with PLAN-Apochromat 63×/1.40 oil DIC M27 objective. For PNA-FISH and immunofluorescence with PNA-FISH at the telomeres, images were taken on a Zeiss 710 laser scanning confocal microscope with PLAN-Apochromat 63×/1.40 oil DIC M27 objective. Images for telomeres where acquired as z stacks at 1 μm steps and a total of 10 steps acquired for each cell.

Telomere-ChIP and Dot blot

Cells were crosslinked in1% formaldehyde in PBS for 10 min at room temperature,125mM glycine was used to quench formaldehyde for 10min. Following, cells were washed twice with PBS and scraped into a tube. Next, 1 ml low salt IP buffer 150mM NaCl, 50mM Tris-Cl(PH7.5), 5mM EDTA, NP-40(0.5%), Triton X-100(1.0%) containing protease inhibitors was used for cell lysis. The lysate was sonicated to shear chromatin. We pre-cleared each sonicated lysates with 30 μL of protein A/G Plus agarose beads before being incubated with 3 μg of antibodies for immunoprecipitation, the mixture of antibody and cell lysates were incubated overnight at 4℃, then 25ul Protein A/G agarose beads was added into the mixture for 2h at 4℃.The beads were washed4 times with 1ml ice cold high salt IP buffer 500mM NaCl, 50mM Tris-Cl(PH7.5), 5mM EDTA, NP-40(0.5%), Triton X-100(1.0%)without protease inhibitors and once with 1ml ice cold low salt IP buffer. The beads were resuspended in 200 ul of resuspension buffer 250mM NaCl, 1mM EDTA, 1%SDS, 1mM Tris-Cl pH8.0 and 0.1mg/mL proteinase K overnight at 56℃.DNA was precipitated by ethanol, and the eluted DNA was transferred to Nylon membrane and hybridized using the Dig-labeled telomere $(TTAGGG)_3$ and Alu repeat probes.

Telomere length measurement assay by Southern blotting

Telomere length was measured by Telo TAGGG Telomere Length Assay Kit (Roche, 12209136001), the procedure was described in the manual. Briefly, genomic DNA was digested by HinfI and RsaI, the products were performed gel electrophoresis and membrane transfer. The membrane was hybridized with Dig-labeled telomere $(TTAGGG)_3$and detected by chemiluminescence system.

Telomere length measurement by QPCR

Telomere length measurement by QPCR was performed as described previously(1).Briefly, Genomic DNA was extracted by DNAeasy blood & tissue kit. All DNA samples were tested in triplicate. The PCR reaction contained 0.75×SYBR Green I, 900nM the telomere primer telg (ACACTAAGGTTTGGGTTTGGGTTTGGGTTTGGGTTAGTGT) and 900nM telc, (TGTTAGGTATCCCTATCCCTATCCCTATCCCTATCCCTAACA). For multiplex QPCR, the PCR reaction was combined with the beta-globin primer pair(final concentrations 500nM each) hbgu (CGGCGGCGGGCGGCGCGGGCTGGGCGGcttcatccacgttcaccttg) and hbgd (GCCCGGCCCGCCGCGCCCGTCCCGCCGgagagaagtctgccgtt). The thermal cycling profile was Stage 1: 15 min at 95℃; Stage 2: 2 cycles of 15 s at 94℃, 15 s at 49℃; and Stage 3: 32 cycles of 15 s at 94℃, 10 s at 62℃, 15 s at 74℃ with

signal acquisition, 10 s at 84℃, 15 s at 88℃ with signal acquisition. The 74℃ reads provided the Ct values for the amplification of the telomere template (in early cycles when the scg signal is still at baseline); the 88℃ reads provided the Ct values for the amplification of the beta-globin template (at this temperature there is no signal from the telomere PCR product, because it is fully melted). Average T/S is expected to be proportional to the average telomere length per cell.

Co-IP

Cells were seeded in 10 cm dish, after 48 hours culture, cells were harvested by trypsinization and nuclear proteins were extracted using 5 volumes of buffer A (10 mM HEPS pH7.5, 10mM KCl, 1.5mM $MgCl_2$, 4mM β-mercaptoethanol) on ice for 20min, centrifuge with 1000g for 10min at 4 ℃. The cell extracts were homogenized 16 strokes (16 up and 16 down) by Teflon pestle driven with a hand-held 0.7hp power drill. Nuclear pellets were suspended in 2.5 volumes of buffer D (20 mM HEPS pH7.5, 420mM KCl, 0.2 mM EDTA, 20% glycerol, 4mM β-mercaptoethanol and protease inhibitors). Then the sample was sonicated briefly and centrifuged 20000g for 10min at 4 ℃. The nuclear extracts were pre-cleared with 25 µL of protein A/G Plus agarose beads before being incubated with antibodies for immunoprecipitation, discard the agarose beads, then the proteins were diluted to 1 µg/µL by using cold PBS. 3 µg of antibodies or IgG were incubated with samples overnight at 4℃, then 25ul Protein A/G agarose beads were added into the mixture for 2h at 4℃.The agarose beads were washed by IP buffer (50mM Tris-HCl, 150 mM NaCl and protease inhibitors) for three times and cold PBS twice. Samples were analyzed by western blot using DSP, TRF1or TRF2 antibodies.

Detection of apoptosis by flow cytometry

Cell apoptosis was measured by Annexin V-FITC Apoptosis Detection Kit (Beyotime).Cells were cultured in a 96 wells microtiter plate at 37℃ and 5% CO_2 in a humidified incubator, 1-5X10^5 cells were collected and washed once with serum-containing media. Cells were re-suspended in 195 µL of 1X Binding Buffer. Then 5 µl of Annexin V-FITC and 10 µl of propidium iodide were also added. Then the cells were incubated at room temperature for 5 min in the dark. Quantification by Flow Cytometry: Analyze Annexin V-FITC binding by flow cytometry (Ex=488 nm; Em=530 nm).

1. Cawthon RM (2009) Telomere length measurement by a novel monochrome multiplex quantitative PCR method. *Nucleic Acids Res* 37(3):e21.
